# Supplementary material for: Analysis of PPARγ Signaling Activity in Psoriasis
Source: Int J Mol Sci. 2021 Aug 10;22(16):8603. doi: 10.3390/ijms22168603 (PMC8395241; doi:10.3390/ijms22168603)
Supplement: Supplementary file 1 [file ijms-22-08603-s001.zip › Supplemental materials_Analysis of PPARg signaling activity in psoriasis/Pathway models/Models images and html files/Model of PPARG signaling in psoriasis/1945.html]

CD14


# Protein CD14

|  |  |
| --- | --- |
| URN | urn:agi-llid:929 |
| Total Entities | 0 |
| Connectivity | 3076 |
| Name | CD14 |
| Description | CD14 molecule |
| Notes | The protein encoded by this gene is a surface antigen that is preferentially expressed on monocytes/macrophages. It cooperates with other proteins to mediate the innate immune response to bacterial lipopolysaccharide. Alternative splicing results in multiple transcript variants encoding the same protein. [provided by RefSeq, Mar 2010] |
| Primary Cell Localization | Plasma membrane |
| Class | Receptor |

---

|  |  |
| --- | --- |
| Pathway | TLR4 Signaling in Leukocytes |
|  | T-Cell Independent B-Cell Activation |
|  | Toll-like Receptors in Sterile Inflammation |
|  | MC1R in anti-Inflammatory Signaling |
|  | Eat me Signal: Apoptotic Cell Initiates Phagocytosis |
|  | Lipids Enhance Apoptotic Cell Engulfment and Reduce Inflammation |
|  | CD16/CD14 Proinflammatory Monocyte Activation |
|  | Macrophage M2-Related Phagocytosis |
|  | AHR Signaling in M1 Macrophages Function |
|  | AXL Receptor Inhibits Macrophages and Dendritic Cells Function |
|  | Neutrophil Recruitment and Priming |
|  | IL6 Expression Targets -> Nociception |
|  | Monoblast -> Promonocyte Surface Expression Markers |
|  | Myeloblast -> Neutrophil Surface Expression Markers |
|  | Myoblast -> Neutrophil Surface Expression Markers |
|  | Hemapoietic Stem Cell -> Lymphoid Multipotent Progenitor Surface Expression Markers |
|  | Proteins Involved in non-Alcoholic Fatty Liver Disease |
|  | Crohn's Disease |
|  | Dendritic Cell Dysfunction in Crohn's disease |
|  | Proteins Involved in Helicobacter Infections |
|  | DupA Induced IL12 Production in Helicobacter Pylori Infection |
|  | Proteins Involved in Ulcerative Colitis |
|  | Antigen-Presenting Cell Role in Asthma |
|  | Monocytes Function in Gout |
|  | Dendritic Cell Dysfunction in Psoriatic Arthritis |
|  | TLR2 Induced Synovial Fibroblast Activation in Rheumatoid Arthritis |
|  | Proteins Involved in HIV Type 1 Infection |
|  | Toll-like Receptors (TLR) in Periodontitis |
|  | Cathepsins Role in Periodontitis |
|  | Proteins Involved in Periodontitis |
|  | Microglia Activation in Alzheimer's Disease |
|  | Microglia Activation in Parkinson's Disease |
|  | Proteins Involved in Parkinson's Disease |
|  | Immune System Role in Otitis Media |
|  | Genes with Mutations Associated with Asthma |
|  | Alveolar Macrophage Negative Regulation Declined in COPD |
|  | Alveolar Macrophages Dysfunction in COPD |
|  | Proteins Involved in Chronic Obstructive Pulmonary Disease |
|  | Toll-like Receptors in Pyelonephritis |
|  | HMGB1 Expression Targets |
|  | Epinephrine/Gs Expression Targets |
|  | Epinephrine/Gq Expression Targets |
|  | Elevated Receptors -> Expression Targets in Adipose Tissue |
|  | Elevated Receptors -> Expression Targets in Joint |
|  | Elevated Receptors -> Expression Targets in Mouth |
|  | Elevated Receptors -> Expression Targets in Colon |
|  | IGF1 -> MEF/MYOD/MYOG Expression Targets |
|  | IGF1 -> STAT Expression Targets |
|  | IGF1 -> ELK/SRF/HIF1A/MYC/SREBF Expression Targets |
|  | IFNG/IFNR Expression Targets |
|  | IL3 Expression Targets |
|  | IL6 Expression Targets |
|  | PGE1 Expression Targets |
|  | CD Markers -> Expression Targets in Lymphoid System and Blood |
|  | GPCRs Family -> Expression Targets in Lymphoid System and Blood |
|  | NOTCH -> Expression Targets in Thymus |
|  | Elevated Receptors -> Expression Targets in Thymus |
|  | CSF1 -> STAT Expression Targets |
|  | KITLG -> STAT Expression Targets |
|  | KITLG -> AP-1/CREB/CREBBP/MYC Expression Targets |
|  | CSF1 -> AP-1/CREB/CREBBP/MYC Expression Targets |
|  | TGFB1-TGFBR2 Expression Targets |
|  | TGFB1-TGFBR1/AP-1 Expression Targets |
|  | TGFB1-ACVRL1 Expression Targets |
|  | TLR4 -> AP-1 Expression Targets |
|  | TLR4 -> AP-1/EGR1/HIF1A Expression Targets |
|  | IL1B -> PGE2 Expression Targets |
|  | IL1B Expression Targets |
|  | TLR4 -> NF-kB/IRF Expression Targets |
|  | TLR4 -> IRF Signaling |
|  | TLR4/5/7/9 -> NF-kB Signaling |
|  | TLR -> AP-1 Signaling |
|  | pprarg neg, uknown targets, ps-positive |
|  | prarg neg,ukn expres targets, ps-positive |
|  | PPARG negative targets |
|  | pparg expr target\_regulators |
|  | Neighbors of disease exacerbation |
|  | Neighbors of tumor progression |
|  | Neighbors of inflammatory status |
|  | Neighbors of overall survival |
|  | Neighbors of disease severity |
|  | Neighbors of mortality |
|  | Neighbors of survival rate |
|  | Neighbors of survival time |
|  | Neighbors of insulin sensitivity |
|  | Neighbors of bacterial load |
|  | Neighbors of graft survival |
|  | Neighbors of lethality |
|  | Neighbors of tumor response |
|  | Neighbors of treatment outcome |
|  | Neighbors of bone mass |
|  | Neighbors of body weight gain |
|  | Neighbors of tumor recurrence |
|  | Neighbors of glucose tolerance |
|  | Neighbors of morbidity |
|  | Neighbors of mortality rate |
|  | Neighbors of viral load |
|  | Neighbors of leukocyte count |
|  | Neighbors of fasting plasma glucose |
|  | Neighbors of gastric cancer risk |
|  | Neighbors of comorbidity |
|  | Neighbors of disease exacerbation |
|  | Neighbors of tumor progression |
|  | Neighbors of inflammatory status |
|  | Neighbors of overall survival |
|  | Neighbors of disease severity |
|  | Neighbors of mortality |
|  | Neighbors of survival rate |
|  | Neighbors of survival time |
|  | Neighbors of insulin sensitivity |
|  | Neighbors of bacterial load |
|  | Neighbors of graft survival |
|  | Neighbors of lethality |
|  | Neighbors of tumor response |
|  | Neighbors of treatment outcome |
|  | Neighbors of bone mass |
|  | Neighbors of body weight gain |
|  | Neighbors of tumor recurrence |
|  | Neighbors of glucose tolerance |
|  | Neighbors of morbidity |
|  | Neighbors of mortality rate |
|  | Neighbors of viral load |
|  | Neighbors of leukocyte count |
|  | Neighbors of fasting plasma glucose |
|  | Neighbors of gastric cancer risk |
|  | Neighbors of comorbidity |
|  | Neighbors of cell development |
|  | Neighbors of immune response |
|  | Neighbors of cell population |
|  | Neighbors of inflammatory response |
|  | Neighbors of angiogenesis |
|  | Neighbors of innate immune response |
|  | Neighbors of cell function |
|  | Neighbors of macrophage activation |
|  | Neighbors of cell formation |
|  | Neighbors of adaptive immune response |
|  | Neighbors of osteoclast development |
|  | Neighbors of epithelial to mesenchymal transition |
|  | Neighbors of immunity |
|  | Neighbors of cell proliferative response |
|  | Neighbors of chemotaxis |
|  | Neighbors of dendritic cell differentiation |
|  | Neighbors of cell survival |
|  | Neighbors of cell phenotype |
|  | Neighbors of T-cell proliferation |
|  | Neighbors of cell adhesion |
|  | Neighbors of T-cell activation |
|  | Neighbors of fibrogenesis |
|  | Neighbors of tumor growth |
|  | Neighbors of dendritic cell development |
|  | Neighbors of cell count |
|  | Neighbors of cell infiltration |
|  | Neighbors of cell interaction |
|  | Neighbors of adipogenesis |
|  | Neighbors of lipid storage |
|  | Neighbors of cell damage |
|  | Neighbors of pregnancy |
|  | Neighbors of monocyte differentiation |
|  | Neighbors of aging |
|  | Neighbors of bone resorption |
|  | Neighbors of adipocyte differentiation |
|  | Neighbors of microglial activation |
|  | Neighbors of neutrophil recruitment |
|  | Neighbors of transendothelial migration |
|  | Neighbors of phagocytosis |
|  | Neighbors of immune system activation |
|  | Neighbors of leukocyte migration |
|  | Neighbors of leukocyte recruitment |
|  | Neighbors of monocyte recruitment |
|  | Neighbors of cell invasion |
|  | Neighbors of ossification |
|  | Neighbors of T-cell function |
|  | Neighbors of autophagy |
|  | Neighbors of monocyte adhesion |
|  | Neighbors of transcription activation |
|  | Neighbors of T-cell response |
|  | Neighbors of tumor immunity |
|  | Neighbors of cell transdifferentiation |
|  | Neighbors of cell growth |
|  | Neighbors of ROS generation |
|  | Neighbors of sensitization |
|  | Neighbors of hemato-encephalic barrier |
|  | Neighbors of macrophage function |
|  | Neighbors of cellular immune response |
|  | Neighbors of colony formation |
|  | Neighbors of macrophage differentiation |
|  | Neighbors of cell differentiation |
|  | Neighbors of life span |
|  | Neighbors of inflammatory disease |
|  | Neighbors of rheumatoid arthritis |
|  | Neighbors of atherosclerosis |
|  | Neighbors of fibrosis |
|  | Neighbors of colitis |
|  | Neighbors of inflammatory bowel disease |
|  | Neighbors of injury |
|  | Neighbors of atherogenesis |
|  | Neighbors of carcinogenesis |
|  | Neighbors of chronic inflammation |
|  | Neighbors of inflammation |
|  | Neighbors of obesity |
|  | Neighbors of diabetes mellitus |
|  | Neighbors of asthma |
|  | Neighbors of pneumonia |
|  | Neighbors of osteoarthritis |
|  | Neighbors of liver fibrosis |
|  | Neighbors of chronic obstructive pulmonary disease |
|  | Neighbors of myocardial infarction |
|  | Neighbors of leukocyte infiltration |
|  | Neighbors of airway inflammation |
|  | Neighbors of hepatitis |
|  | Neighbors of insulin resistance |
|  | Neighbors of infection |
|  | Neighbors of macrophage infiltration |
|  | Neighbors of liver injury |
|  | Neighbors of neuroinflammation |
|  | Neighbors of sepsis |
|  | Neighbors of death |
|  | Neighbors of type 1 diabetes |
|  | Neighbors of neutrophil infiltration |
|  | Neighbors of heart disease |
|  | Neighbors of lung injury |
|  | Neighbors of immunopathology |
|  | Neighbors of autoimmunity |
|  | Neighbors of acute lung injury |
|  | Neighbors of gastric cancer |
|  | Neighbors of experimental autoimmune encephalomyelitis |
|  | Neighbors of colorectal cancer |
|  | Neighbors of breast cancer |
|  | Neighbors of ischemia |
|  | Neighbors of systemic lupus erythematosus |
|  | Neighbors of kidney disease |
|  | Neighbors of heart failure |
|  | Neighbors of cancer |
|  | Neighbors of cerebral ischemia |
|  | Neighbors of neoplasm |
|  | Neighbors of coronary artery disease |
|  | Neighbors of endothelial cell dysfunction |
|  | Neighbors of liver disease |
|  | Neighbors of hypertension |
|  | Neighbors of allograft rejection |
|  | Neighbors of epithelial cell |
|  | Neighbors of vascular smooth muscle cell |
|  | Neighbors of umbilical vein endothelial cell |
|  | Neighbors of monocyte |
|  | Neighbors of macrophage |
|  | Neighbors of adipocyte |
|  | Neighbors of activated monocyte |
|  | Neighbors of airway epithelial cell |
|  | Neighbors of PBMC |
|  | Neighbors of microglia |
|  | Neighbors of intestine epithelium cell |
|  | Neighbors of hepatocyte |
|  | Neighbors of endothelial cell |
|  | Neighbors of dendritic cell |
|  | Neighbors of Kuppfer cell |
|  | Neighbors of inflammatory cell |
|  | Neighbors of neutrophil |
|  | Neighbors of astrocyte |
|  | Neighbors of immunocompetent cell |
|  | Neighbors of granulocyte |
|  | Neighbors of myeloid cell |
|  | Neighbors of stem cell |
|  | Neighbors of leukocyte |
|  | Neighbors of mammary epithelial cell |
|  | Neighbors of peripheral lymphocyte |
|  | Neighbors of lung |
|  | Neighbors of liver |
|  | Neighbors of kidney |
|  | Neighbors of skin |
|  | Neighbors of intestine |
|  | Neighbors of airway |
|  | Neighbors of blood vessel |
|  | Neighbors of aorta |
|  | Neighbors of gastrointestinal tract |
|  | Neighbors of carotid artery |
|  | Neighbors of synovial membrane |
|  | Neighbors of retina |
|  | Neighbors of pancreas |
|  | Neighbors of lymphatic system |
|  | Neighbors of ankle joint |
|  | Neighbors of vitreous body |
|  | Neighbors of oxidized LDL |
|  | Neighbors of JNK |
|  | Neighbors of PPAR |
|  | Neighbors of Jun/Fos |
|  | Neighbors of PKC |
|  | Neighbors of PI3K |
|  | Neighbors of STAT family |
|  | Neighbors of IFNAR ligand |
|  | Neighbors of cytokine |
|  | Neighbors of interferon |
|  | Neighbors of ERK1/2 |
|  | Neighbors of PKA |
|  | Neighbors of IgG |
|  | Neighbors of HDL |
|  | Neighbors of histone deacetylase |
|  | Neighbors of GPCR |
|  | Neighbors of Notch |
|  | Neighbors of heat shock protein 90 |
|  | Neighbors of lectin |
|  | Neighbors of histone H3 |
|  | Neighbors of retinoid-X receptor subfamily |
|  | Neighbors of TORC1 |
|  | Neighbors of angiotensin receptor |
|  | Neighbors of RA receptor |
|  | Neighbors of neuropeptides |
|  | Model of PPARG signaling in psoriasis |
|  | Overview. Diseases of the respiratory (chapter 9) |
|  | 1\_The role of Toll-like receptors in pyelonephritis |
|  | 3\_Role of alveolar macrophages in pulmonary tissue destruction in COPD |
|  | 1\_Th2 cell response in asthma |
|  | 1\_Defects in response to pathogens in the gut promotes inflammation in Crohn's disease |
|  | 4\_1\_Polymorphisms associated with inflammatory bowel diseases |

---

|  |  |
| --- | --- |
| Group | Genes Associated with Ulcerative Colitis |
|  | Genes with Mutations Associated with Asthma |

---

|  |  |
| --- | --- |
| MedScan ID | 929 |

---

|  |  |
| --- | --- |
| LocusLink ID | 929 |
|  | 12475 |
|  | 60350 |

---

|  |  |
| --- | --- |
| Alias | Myeloid cell-specific leucine-rich glycoprotein |
|  | OTTHUMP00000223710 |
|  | CD14 |
|  | monocyte surface glycoprotein CD14 |
|  | Monocyte differentiating antigen CD14 |
|  | CD14 antigen |
|  | CD14\_HUMAN |
|  | Monocyte differentiation antigen CD14 |
|  | OTTHUMP00000223711 |
|  | antigen cd14 |
|  | CD14 molecule |
|  | Monocyte differentiation antigen CD14 precursor |
|  | Monocyte differentiating antigen CD14 precursor |
|  | LPS-R |
|  | monocyte surface glycoprotein CD14 precursor |

---

|  |  |
| --- | --- |
| GO ID | 0071723 |
|  | 0001530 |
|  | 0070891 |
|  | 0001847 |
|  | 0016019 |
|  | 0007249 |
|  | 0002755 |
|  | 0002756 |
|  | 0035666 |
|  | 0006915 |
|  | 0097190 |
|  | 0007166 |
|  | 0071726 |
|  | 0071222 |
|  | 0071223 |
|  | 0071219 |
|  | 0071727 |
|  | 0006954 |
|  | 0045087 |
|  | 0031663 |
|  | 0070266 |
|  | 0034128 |
|  | 0043312 |
|  | 0006909 |
|  | 1901224 |
|  | 0050715 |
|  | 0045807 |
|  | 0032729 |
|  | 2000484 |
|  | 0032760 |
|  | 0032481 |
|  | 0006898 |
|  | 0051602 |
|  | 0045471 |
|  | 0009408 |
|  | 0032026 |
|  | 0034612 |
|  | 0034142 |
|  | 0038123 |
|  | 0038124 |
|  | 0002224 |
|  | 0005794 |
|  | 0031362 |
|  | 0009986 |
|  | 0010008 |
|  | 0009897 |
|  | 0070062 |
|  | 0005576 |
|  | 0005615 |
|  | 0046696 |
|  | 0045121 |
|  | 0005886 |
|  | 0030667 |
|  | 0002376 |
|  | 0009617 |
|  | 0002237 |
|  | 0031225 |
|  | 0016020 |
|  | 0032496 |
|  | 0034134 |
|  | 0034138 |
|  | 0008063 |
|  | 0006950 |
|  | 0005515 |
|  | 0006955 |
|  | 0070391 |
|  | 0019898 |
|  | 0048503 |

---

|  |  |
| --- | --- |
| KEGG ID | hsa:929 |
|  | mmu:12475 |
|  | rno:60350 |

---

|  |  |
| --- | --- |
| MedLine Reference | 10395652 |
|  | 10575214 |
|  | 11076863 |
|  | 11083760 |
|  | 11145656 |
|  | 11489942 |
|  | 11745332 |
|  | 11779220 |
|  | 11810121 |
|  | 11825494 |
|  | 11841490 |
|  | 11854210 |
|  | 11876746 |
|  | 11932927 |
|  | 11935032 |
|  | 12011764 |
|  | 12067299 |
|  | 12106839 |
|  | 12117913 |
|  | 12140663 |
|  | 12171914 |
|  | 12174084 |
|  | 12196527 |
|  | 12207338 |
|  | 12324469 |
|  | 12410798 |
|  | 12417309 |
|  | 12438323 |
|  | 12477932 |
|  | 12594207 |
|  | 12624278 |
|  | 12626456 |
|  | 12663765 |
|  | 12679473 |
|  | 12700637 |
|  | 12731067 |
|  | 12754215 |
|  | 12757265 |
|  | 12825176 |
|  | 12832426 |
|  | 12853157 |
|  | 12897754 |
|  | 12911501 |
|  | 12938192 |
|  | 12942028 |
|  | 12960274 |
|  | 14500479 |
|  | 14508193 |
|  | 14517492 |
|  | 14648231 |
|  | 14672339 |
|  | 14673018 |
|  | 14706103 |
|  | 15034063 |
|  | 15069085 |
|  | 15080833 |
|  | 15081257 |
|  | 15116260 |
|  | 15120158 |
|  | 15166925 |
|  | 15257175 |
|  | 15269840 |
|  | 15358661 |
|  | 15373760 |
|  | 15378299 |
|  | 15489334 |
|  | 15489336 |
|  | 15491315 |
|  | 15558232 |
|  | 15591473 |
|  | 15602630 |
|  | 15619435 |
|  | 15655821 |
|  | 15670766 |
|  | 15714129 |
|  | 15753758 |
|  | 15764151 |
|  | 15778383 |
|  | 15842262 |
|  | 15932345 |
|  | 15946916 |
|  | 15975149 |
|  | 16038043 |
|  | 16061600 |
|  | 16101942 |
|  | 16142747 |
|  | 16165702 |
|  | 16239565 |
|  | 16266379 |
|  | 16273620 |
|  | 16273622 |
|  | 16275943 |
|  | 16283111 |
|  | 16290232 |
|  | 16335952 |
|  | 16344560 |
|  | 16368002 |
|  | 16381901 |
|  | 16387800 |
|  | 16437636 |
|  | 16443672 |
|  | 16444757 |
|  | 16467036 |
|  | 16473828 |
|  | 16492974 |
|  | 16552356 |
|  | 16611358 |
|  | 16625214 |
|  | 16706818 |
|  | 16751411 |
|  | 16785528 |
|  | 16848791 |
|  | 16860318 |
|  | 16933467 |
|  | 16950521 |
|  | 17003960 |
|  | 17007028 |
|  | 17046070 |
|  | 17052752 |
|  | 17086894 |
|  | 17098305 |
|  | 17126402 |
|  | 17205552 |
|  | 17217435 |
|  | 17259806 |
|  | 17274987 |
|  | 17304102 |
|  | 17309585 |
|  | 17436151 |
|  | 17438094 |
|  | 17448042 |
|  | 17471431 |
|  | 17515856 |
|  | 17565650 |
|  | 17600225 |
|  | 17617027 |
|  | 2448876 |
|  | 2453848 |
|  | 2462937 |
|  | 2472171 |
|  | 2779588 |
|  | 3385210 |
|  | 7583357 |
|  | 8798531 |
|  | 9142045 |
|  | 9548256 |
|  | 9562334 |
|  | 9655682 |
|  | 10047547 |
|  | 10202023 |
|  | 10221645 |
|  | 10349636 |
|  | 10679400 |
|  | 11035061 |
|  | 11042159 |
|  | 11076861 |
|  | 11123339 |
|  | 11217851 |
|  | 11254735 |
|  | 11796577 |
|  | 11870620 |
|  | 12146799 |
|  | 12165539 |
|  | 12239221 |
|  | 12244136 |
|  | 12244198 |
|  | 12370383 |
|  | 12391013 |
|  | 12424240 |
|  | 12427659 |
|  | 12466851 |
|  | 12482856 |
|  | 12496149 |
|  | 12639839 |
|  | 12935356 |
|  | 12964032 |
|  | 14516777 |
|  | 14517279 |
|  | 14614560 |
|  | 14742577 |
|  | 14752288 |
|  | 15034566 |
|  | 15142274 |
|  | 15194867 |
|  | 15208271 |
|  | 15240706 |
|  | 15271679 |
|  | 15306695 |
|  | 15322035 |
|  | 15331624 |
|  | 15459483 |
|  | 15522312 |
|  | 15541715 |
|  | 15557205 |
|  | 15585870 |
|  | 15611337 |
|  | 15644310 |
|  | 15661914 |
|  | 15724245 |
|  | 15731076 |
|  | 15755524 |
|  | 15795240 |
|  | 15814723 |
|  | 15895089 |
|  | 15896296 |
|  | 15941778 |
|  | 16081822 |
|  | 16141072 |
|  | 16141073 |
|  | 16148141 |
|  | 16163057 |
|  | 16188968 |
|  | 16204620 |
|  | 16210672 |
|  | 16225933 |
|  | 16229899 |
|  | 16237099 |
|  | 16428731 |
|  | 16439470 |
|  | 16480837 |
|  | 16574244 |
|  | 16705022 |
|  | 1694207 |
|  | 16980981 |
|  | 16995947 |
|  | 17039565 |
|  | 17167049 |
|  | 17283106 |
|  | 17292937 |
|  | 17384086 |
|  | 17456769 |
|  | 17474083 |
|  | 2467257 |
|  | 7532683 |
|  | 8612135 |
|  | 8706013 |
|  | 8921956 |
|  | 9127007 |
|  | 9353071 |
|  | 9665271 |
|  | 10854787 |
|  | 12046090 |
|  | 12113681 |
|  | 12218159 |
|  | 12632533 |
|  | 14572767 |
|  | 14599981 |
|  | 15153652 |
|  | 15310678 |
|  | 16879219 |
|  | 8598386 |
|  | 9201265 |
|  | 9784508 |

---

|  |  |
| --- | --- |
| CellType | squamous epithelial cells |
|  | urothelial cells |
|  | smooth muscle cells |
|  | cells in endometrial stroma |
|  | cells in red pulp |
|  | glandular cells |
|  | cells in white pulp |
|  | decidual cells |
|  | Macrophages |
|  | trophoblastic cells |
|  | Pneumocytes |
|  | germinal center cells |
|  | myocytes |
|  | Purkinje Cells |
|  | cells in seminiferus ducts |
|  | non-germinal center cells |
|  | glial cells |
|  | epidermal cells |
|  | Leydig Cells |
|  | respiratory epithelial cells |
|  | neuronal cells |
|  | cells in glomeruli |
|  | follicle cells |
|  | cells in tubules |
|  | ovarian stroma cells |
|  | lymphoid tissue |
|  | exocrine glandular cells |
|  | hematopoietic cells |
|  | islets of Langerhans |
|  | bile duct cells |
|  | Hepatocytes |
|  | cells in granular layer |
|  | cells in molecular layer |

---

|  |  |
| --- | --- |
| Tissue | tonsil |
|  | urinary bladder |
|  | smooth muscle |
|  | uterus, post-menopause |
|  | spleen |
|  | parathyroid gland |
|  | Placenta |
|  | Lung |
|  | uterus, pre-menopause |
|  | stomach, lower |
|  | gall bladder |
|  | stomach, upper |
|  | Prostate |
|  | lymph node |
|  | heart muscle |
|  | Cerebellum |
|  | vagina |
|  | testis |
|  | rectum |
|  | hippocampus |
|  | cerebral cortex |
|  | vulva/anal skin |
|  | Salivary gland |
|  | nasopharynx |
|  | thyroid gland |
|  | seminal vesicle |
|  | oral mucosa |
|  | Kidney |
|  | cervix, uterine |
|  | Adrenal gland |
|  | skeletal muscle |
|  | Ovary |
|  | Appendix |
|  | Skin |
|  | lateral ventricle |
|  | colon |
|  | small intestine |
|  | Pancreas |
|  | duodenum |
|  | bone marrow |
|  | Liver |
|  | epididymis |
|  | breast |
|  | esophagus |
|  | bronchus |
|  | fallopian tube |

---

|  |  |
| --- | --- |
| Organism | Homo sapiens {Organism urn:agi-taxid:9606} |
|  | Mus musculus {Organism urn:agi-taxid:10090} |
|  | Rattus norvegicus {Organism urn:agi-taxid:10116} |
|  | Homo sapiens |
|  | Mus musculus |
|  | Rattus norvegicus |

---

|  |  |
| --- | --- |
| Mouse chromosome position | 18 19.46 cM |
|  | 18 31.0 cM |

---

|  |  |
| --- | --- |
| OMIM ID | 158120 |

---

|  |  |
| --- | --- |
| Rat chromosome position | 18p11 |

---

|  |  |
| --- | --- |
| Hugo ID | 1628 |
|  | HGNC:1628 |

---

|  |  |
| --- | --- |
| Human chromosome position | 5q31.3 |
|  | 5q31.1 |
|  | 5q22-q32 |

---

|  |  |
| --- | --- |
| Swiss-Prot Accession | P08571 |
|  | P08571.2 |
|  | P10810 |
|  | Q3UB18 |
|  | Q3UE54 |
|  | Q4FJP7 |
|  | P10810.1 |
|  | Q63691 |
|  | Q63691.2 |
|  | Q53XT5 |
|  | Q96FR6 |
|  | Q96L99 |
|  | Q9UNS3 |
|  | Q6GT04 |
|  | Q3TE16 |
|  | Q3U6A3 |
|  | Q91V69 |
|  | Q91VC2 |
|  | Q91VD3 |
|  | Q920X7 |
|  | Q920X8 |
|  | Q920X9 |
|  | O88955 |

---

|  |  |
| --- | --- |
| PIR ID | A27637 |
|  | S03605 |

---

|  |  |
| --- | --- |
| GenBank ID | NC\_000005 |
|  | NM\_001174104 |
|  | NP\_001167575 |
|  | NM\_001174105 |
|  | NP\_001167576 |
|  | NM\_001040021 |
|  | NP\_001035110 |
|  | NM\_000591 |
|  | NP\_000582 |
|  | NG\_023178 |
|  | AC116353 |
|  | CH471062 |
|  | EAW62037 |
|  | HQ199230 |
|  | ADX31876 |
|  | U00699 |
|  | AAA20640 |
|  | X06882 |
|  | CAA29999 |
|  | AB446505 |
|  | BAG55282 |
|  | AF097942 |
|  | AAC83816 |
|  | AK313277 |
|  | BAG36085 |
|  | AY044269 |
|  | AAL02401 |
|  | BC010507 |
|  | AAH10507 |
|  | BP300412 |
|  | BT007331 |
|  | AAP35995 |
|  | CF593452 |
|  | DA269805 |
|  | DA426297 |
|  | DA681405 |
|  | M86511 |
|  | AAA51930 |
|  | W74441 |
|  | X13334 |
|  | CAA31711 |
|  | P08571 |
|  | NC\_000084 |
|  | NM\_009841 |
|  | NP\_033971 |
|  | AB039054 |
|  | BAB68578 |
|  | AB039056 |
|  | BAB68580 |
|  | AB039057 |
|  | BAB68581 |
|  | AB039058 |
|  | BAB68582 |
|  | AB039059 |
|  | BAB68583 |
|  | AB039060 |
|  | BAB68584 |
|  | AB039061 |
|  | BAB68585 |
|  | AB039062 |
|  | BAB68586 |
|  | AC027740 |
|  | CH466557 |
|  | EDK97172 |
|  | D10912 |
|  | M34510 |
|  | AAA37387 |
|  | X13987 |
|  | CAA32166 |
|  | AK149744 |
|  | BAE29057 |
|  | AK151139 |
|  | BAE30146 |
|  | AK152427 |
|  | AK153229 |
|  | BAE31822 |
|  | AK156262 |
|  | BAE33646 |
|  | AK169880 |
|  | BAE41432 |
|  | BC057889 |
|  | AAH57889 |
|  | BY498446 |
|  | CT010355 |
|  | CAJ18563 |
|  | EH108279 |
|  | X13333 |
|  | CAA31710 |
|  | P10810 |
|  | NC\_005117 |
|  | XM\_006254603 |
|  | XP\_006254665 |
|  | NM\_021744 |
|  | NP\_068512 |
|  | AC\_000086 |
|  | AAHX01094551 |
|  | AC125248 |
|  | AF087944 |
|  | AAC35372 |
|  | CH473974 |
|  | EDL76309 |
|  | AF087943 |
|  | AAC35371 |
|  | BC061733 |
|  | AAH61733 |
|  | FQ219480 |
|  | U51804 |
|  | AAB01154 |
|  | Q63691 |
|  | NC\_018916 |
|  | AMYH02012340 |
|  | AM392692 |
|  | CAL37570 |
|  | AM392824 |
|  | CAL37702 |
|  | AM393386 |
|  | CAL38264 |
|  | AM393697 |
|  | CAL38573 |
|  | AC\_000040 |
|  | AAHY01137757 |
|  | AC\_000137 |
|  | ABBA01026424 |
|  | AB039055 |
|  | BAB68579 |
|  | NT\_029289 |
|  | NW\_001838953 |
|  | NW\_922784 |
|  | AC\_000048 |
|  | CR590334 |
|  | CR590355 |
|  | CR590525 |
|  | CR591451 |
|  | CR593752 |
|  | CR594268 |
|  | CR595571 |
|  | CR598974 |
|  | CR600557 |
|  | CR600702 |
|  | CR601474 |
|  | CR602302 |
|  | CR603280 |
|  | CR603424 |
|  | CR605705 |
|  | CR609600 |
|  | CR610580 |
|  | CR610684 |
|  | CR612020 |
|  | CR613552 |
|  | CR614015 |
|  | CR614726 |
|  | CR616071 |
|  | CR616612 |
|  | CR617446 |
|  | CR620138 |
|  | CR620234 |
|  | CR620555 |
|  | CR626311 |
|  | CR626579 |
|  | NT\_039674 |
|  | NW\_001030631 |
|  | Q3TE16 |
|  | Q3U6A3 |
|  | Q3UB18 |
|  | Q3UE54 |
|  | Q4FJP7 |
|  | Q91V69 |
|  | Q91VC2 |
|  | Q91VD3 |
|  | Q920X7 |
|  | Q920X8 |
|  | Q920X9 |
|  | NW\_001084735 |
|  | NW\_047511 |
|  | O88955 |
|  | Q53XT5 |
|  | 117644414 |
|  | 117645596 |
|  | 117646212 |
|  | 117646910 |
|  | 119582441 |
|  | 121946438 |
|  | 14714729 |
|  | 15667229 |
|  | 180021 |
|  | 20141203 |
|  | 29741 |
|  | 30583501 |
|  | 312399 |
|  | 3983127 |
|  | 74754961 |
|  | Q0JSE1 |
|  | 115957 |
|  | 123783222 |
|  | 123784372 |
|  | 123790973 |
|  | 123794411 |
|  | 123797626 |
|  | 148664756 |
|  | 15822854 |
|  | 15822856 |
|  | 15822858 |
|  | 15822860 |
|  | 15822862 |
|  | 15822864 |
|  | 15822866 |
|  | 15822868 |
|  | 15822870 |
|  | 2342527 |
|  | 34784392 |
|  | 387121 |
|  | 50339 |
|  | 71060039 |
|  | 74139974 |
|  | 74185341 |
|  | 74199487 |
|  | 74207846 |
|  | 74220943 |
|  | 81867324 |
|  | 81867331 |
|  | 81867332 |
|  | 81867579 |
|  | 81867580 |
|  | 81867581 |
|  | 1262920 |
|  | 146345387 |
|  | 149017258 |
|  | 3599525 |
|  | 3599527 |
|  | 38197682 |
|  | 81861565 |

---

|  |  |
| --- | --- |
| Swiss-Prot ID | CD14\_HUMAN |
|  | CD14\_MOUSE |
|  | CD14\_RAT |
|  | Q4FJP7\_MOUSE |

---

|  |  |
| --- | --- |
| Cell Localization | Cell membrane |
|  | Secreted |
|  | Membrane raft |
|  | Golgi apparatus |
|  | Plasma membrane |

---

|  |  |
| --- | --- |
| Ensembl ID | ENSG00000170458 |
|  | ENSP00000304236.6 |
|  | ENST00000302014.11 |
|  | ENSP00000385519.2 |
|  | ENST00000401743.6 |
|  | ENSMUSG00000051439 |
|  | ENSMUSP00000056669.6 |
|  | ENSMUST00000061829.7 |
|  | ENSRNOG00000017819 |
|  | ENSRNOP00000023977.3 |
|  | ENSRNOT00000023977.6 |
|  | ENSP00000304236 |
|  | ENST00000302014 |
|  | ENSP00000385519 |
|  | ENST00000401743 |
|  | ENSMUSP00000056669 |
|  | ENSMUST00000061829 |
|  | ENSRNOP00000023977 |
|  | ENSRNOT00000023977 |

---

|  |  |
| --- | --- |
| MGI ID | MGI:88318 |
|  | 88318 |

---

|  |  |
| --- | --- |
| RGD ID | 620588 |

---

|  |  |
| --- | --- |
| Unigene ID | Hs.163867 |
|  | Mm.3460 |
|  | Rn.42942 |
|  | Hs.638333 |

---

|  |  |
| --- | --- |
| Homologene ID | 493 |

---

|  |  |
| --- | --- |
| Shape | Stick-vertex |

---

|  |  |
| --- | --- |
| IPI ID | IPI00029260 |
|  | IPI00308990 |
|  | IPI00231949 |

---

|  |  |
| --- | --- |
| Homo sapiens Chromosome position | 5q22-q32 |

---

|  |  |
| --- | --- |
| Ensembl Transcript ID | ENST00000302014 |
|  | ENST00000401743 |

---

|  |  |
| --- | --- |
| FunctionalClass | GPI anchor binding |
|  | peptidoglycan receptor |
|  | protein binding |

---

|  |  |
| --- | --- |
| Mus musculus Chromosome position | 18 31.0 cM |

---

|  |  |
| --- | --- |
| KEGG pathway | Hematopoietic cell lineage |
|  | MAPK signaling pathway |
|  | Regulation of actin cytoskeleton |
|  | Toll-like receptor signaling pathway |

---

|  |  |
| --- | --- |
| Rattus norvegicus Chromosome position | 18p11 |

---

|  |  |
| --- | --- |
| Ensembl Gene ID | ENSG00000170458 |

---
